# Predicting drug resistance related to ABC transporters using unsupervised Consensus Self-Organizing Maps

**DOI:** 10.1038/s41598-018-25235-9

**Published:** 2018-05-01

**Authors:** Roger Estrada-Tejedor, Gerhard F. Ecker

**Affiliations:** 1 IQS School of Engineering, Universitat Ramon Llull, Via Augusta 390, 08017 Barcelona, Spain; 2 University of Vienna, Department of Pharmaceutical Chemistry, Althanstrasse 14, 1090 Vienna, Austria

## Abstract

ATP binding cassette (ABC) transporters play a pivotal role in drug elimination, particularly on several types of cancer in which these proteins are overexpressed. Due to their promiscuous ligand recognition, building computational models for substrate classification is quite challenging. This study evaluates the use of modified Self-Organizing Maps (SOM) for predicting drug resistance associated with P-gp, MPR1 and BCRP activity. Herein, we present a novel multi-labelled unsupervised classification model which combines a new clustering algorithm with SOM. It significantly improves the accuracy of substrates classification, catching up with traditional supervised machine learning algorithms. Results can be applied to predict the pharmacological profile of new drug candidates during the drug development process.

## Introduction

ATP binding cassette transporters (ABC-transporters) are ubiquitous integral membrane proteins involved in the active transport of ligands across biological membranes, using the energy of ATP hydrolysis. They are critical determinants of bioavailability, distribution, and elimination of metabolites and xenobiotics. Furthermore, ABC-transporters have been recognized as being responsible for drug resistance in tumour therapy^1,2^. Particularly, P-glycoprotein (P-gp), multidrug resistance protein 1 (MPR1) and breast cancer resistance protein (BCRP) are overexpressed in several cancers^3–6^. They are considered being the most relevant ABC-transporters in conferring a multidrug-resistance phenotype to cancer cells^1^. All three transporters are characterized by a broad, partly overlapping substrate profile. While P-gp and BCRP predominantly transport neutral and positively charged compounds across cell membranes, MRP1 is annotated with negatively charged compounds and glutathione conjugates.

Interaction between ABC-transporters and drug candidates highly determine their pharmacological profile^7^. Thus, it is of great interest to predict the effect of ABC-transporters on a given chemical compound. Although several *in silico* models have been reported for predicting substrates and inhibitors for these proteins, these were binary classification models applied on balanced datasets^8–11^. Noteworthy, best performance was achieved when using supervised learning methods such as random forest^12^. However, these approaches suffer from two drawbacks. First, in real life scenarios data sets are highly imbalanced. Second, supervised methods are prone to bias introduced by the method used. For getting unbiased prediction models, the application of unsupervised methods could be extremely useful.

Self-Organizing Maps are unsupervised neural networks based on competitive learning in which a dataset, originally described in a high dimensional space, is projected onto a two-dimensional space applying a topology preserving mapping. Each SOM position accounts for a neuron, with a randomly initialized weight vector. During the training procedure, neurons compete to determine the winning neuron for each object presented by means of a similarity measure between input values and neural weights. The weights of the winning neuron and its neighbours are adjusted before the next object is presented to the neural net. It is important to note that no information about the class label is used throughout the learning process.

Combined with lazy classification algorithms such as k Nearest Neighbour (k-NN), SOMs can be easily adapted as a classification method. Unfortunately, our results evince that the combination of SOM and k-NN methods has difficulties in dealing with imbalanced data, even when applying oversampling. For this reason, we propose a new algorithm (CSOM) to predict P-gp, MRP1 and BCRP substrates based on the probability of each SOM coordinate to belong to every class.

As the accuracy of the results obtained highly depends on the attributes used for the description of the input examples, several sets of molecular descriptors have been considered. The novel CSOM methodology has been validated with well-stablished imbalanced datasets and proved to be useful for the classification of imbalanced data sets. It improved the results obtained by k-NN and allowed to classify P-gp, MRP1 and BCRP substrates.

## Methods

### Data sets

Experimental information regarding P-gp, MRP1 and BCRP substrates was obtained from the screening data reported by Szakács *et al*.^13^. This database contains the Pearson’s correlation coefficient between the cytotoxicity of more than 1400 compounds (in a panel of 60 cancer cell lines) and the mRNA levels of 48 known human ABC transporters. The original data set was carefully curated in previous studies leading to a final training set of 1204 compounds^9^. Molecules with correlation coefficients lower than −0.3 were considered substrates for the transporters of interest. This threshold yields to a high imbalanced data set containing a total of 190 substrates for P-gp (77), MRP1 (66) and BCRP (47) transporters and 1012 compounds classified as non-substrates.

Unfortunately, the data set only contains two compounds able to act as substrate for more than one transporter. This situation hampers the possibility to obtain a model to predict dual or triple interactions involving P-gp, MRP1 and BCRP transporters.

Four benchmark data sets obtained from publicly available data set repositories (UCI Machine Learning Repository^14^ and KEEL^15^) were used for the evaluation of the new algorithm. All considered databases contained multi-class imbalanced data sets (Table 1).

**Table 1:** Description of the benchmark data sets used for the validation of the model.

Data	Inputs	Attributes	Classes	Imbalance ratio
Wines	178	13	3	1:2
New Thyroid	193	5	3	1:19
Cars	1728	6	4	1:19
Yeast	1484	8	10	1:93

### Molecular Descriptors

Previous studies revealed that subdivided surface areas (VSA) and atom count descriptors have a high influence on the identification of substrates for ABC-transporters when applying binary classifiers^16^. Furthermore, lipophilicity and molecular size were identified as crucial for P-gp and BCRP substrates classifications, whereas partial charge-related descriptors played an important role for the identification of MRP1 substrates^16^. Noteworthy, these sets of descriptors account for independent information outlining the features that characterize the substrates for each individual target. Thus, we considered them as a starting point to identify the key descriptors for multi-classification. Correlated descriptors with Pearson’s correlation coefficient greater than 0.9 were deleted, obtaining a final set of 17 descriptors (DD17, Table 2). All descriptors were calculated using the MOE 2014.09 software package^17^. Prior to descriptor calculation, all molecules were neutralized, energy minimized and their partial charges were calculated.

**Table 2:** Descriptors reported by Demel *et al*.^16^ for the binary classification of P-gp, MRP1 and BCRP substrates.

Model	Selected Descriptors
P-gp	apol, chi0_C, chi0v_C, chi1_C, rings, PEOE_VSA-5, PEOE_VSA_POL, PEOE_VSA_PPOS, SlogP_VSA0, SMR_VSA2, TPSA, opr_brigid
MRP1	a_count, a_hyd, chi1v, opr_nring, PEOE_VSA + 3, PEOE_VSA + 5, PEOE_VSA-4, PEOE_VSA-6, Q_VSA_PNEG, vsa_acc
BCRP	a_count, a_hyd, a_nC, a_nH, chi1v, SlogP_VSA1, SlogP_VSA2, SlogP_VSA8, SMR_VSA1, SMR_VSA6, VDistMa
DD17	apol, opr_brigid, PEOE_VSA + 3, PEOE_VSA + 5, PEOE_VSA-4, PEOE_VSA-5, PEOE_VSA-6, PEOE_VSA_POL, Q_VSA_PNEG, SlogP_VSA0, SlogP_VSA1, SlogP_VSA2, SlogP_VSA8, SMR_VSA1, SMR_VSA2, SMR_VSA6, vsa_acc

A total of 17 descriptors with Pearson’s correlation coefficient lower than 0.9 were identified from all models and they were joined into the DD17 set.

We hypothesized that the combination of uncorrelated descriptors derived from binary classifiers would be able to distinguish between P-gp, MRP1 and BCRP substrates. However, being aware of the pivotal role that descriptors play on the model’s performance, we inquired into the need of including other descriptors (by using dimensionality reduction algorithms) to achieve good accuracies. One completely different set of descriptors are those derived from the ChemGPS-NP service. These are composed by 8 principal components^18^ derived from a distance matrix to satellite compounds, with the first four PCs accounting for 77% of the total variance. They mainly reflect the size, the aromaticity, the lipophilicity and the flexibility of compounds.

We also proposed the use of Auto-Associative Neural Networks (AANN) as an alternative to include non-linear relationships between the original molecular descriptors and the calculated features. A 1-hidden neural network was applied on a set of 67 1D/2D descriptors calculated in MOE (a graphical representation of AANN is included in Fig. S1 in the supporting information). We considered only descriptors with physicochemical meaning, excluding atom count, fractional descriptors and drug-like filters. Descriptors were scaled using a sigmoidal function before applying Single Value Decomposition to predict the best weights for reducing the feature space to 10 variables^19^.

Finally, looking for more generic descriptors, Shannon Entropy Descriptor (SHED) fingerprints were used. SHED fingerprints contain 10 values that account for the variability of a 10 feature pair distribution (obtained by the combination of acceptor, donor, hydrophobic and aromatic atoms) by considering Shannon entropy^20^.

### Dealing with imbalanced data

Many strategies have been reported in literature to handle imbalanced data by modifying the data set (acting on sampling) or the classifier (by applying cost-sensitive methods).

In the attempt to balance the data set, two main strategies are usually considered: reducing the number of examples in the majority class by cherry-picking (random undersampling), or increasing the population of minority classes (oversampling). In the latter case, classes with fewer examples can be enlarged by replication (random re-sampling) or by creating new examples. We applied the Synthetic Minority Oversampling TEchnique (SMOTE) for over-sampling, which is based on the generation of synthetic examples near to the minority class samples in the feature space^21^.

The accuracy metrics broadly used for classification are commonly sensitive to the number of the examples^22^, which is not appropriate for handling imbalanced data. Thus, choosing a suitable metric for imbalanced data sets is crucial in order to reflect the behaviour of minority classes and to avoid unrealistic good accuracies due to the majority class. Since recall values are not sensitive to the number of examples, its arithmetic and geometric mean have been successfully applied in multi-class imbalanced data sets^23^. Additionally, in order to combine recall and precision in one single value, we used the F measure, which corresponds to the harmonic mean of precision and recall^24^.

### Consensus Self-Organizing Maps (CSOM)

Self-Organizing Maps must be combined with a clustering or other classification method that allow to assign the class of a given example when it is mapped into SOM coordinates. In this study we propose a new strategy named CSOM and compare its performance with a k-NN classifier. As most of the misclassifications are related with points located near class boundaries, information gathered in the border region needs to be taken into account. For this we propose an algorithm based on weighted voting to calculate the probability of an unoccupied coordinate to belong to each class.

**Figure 1:**
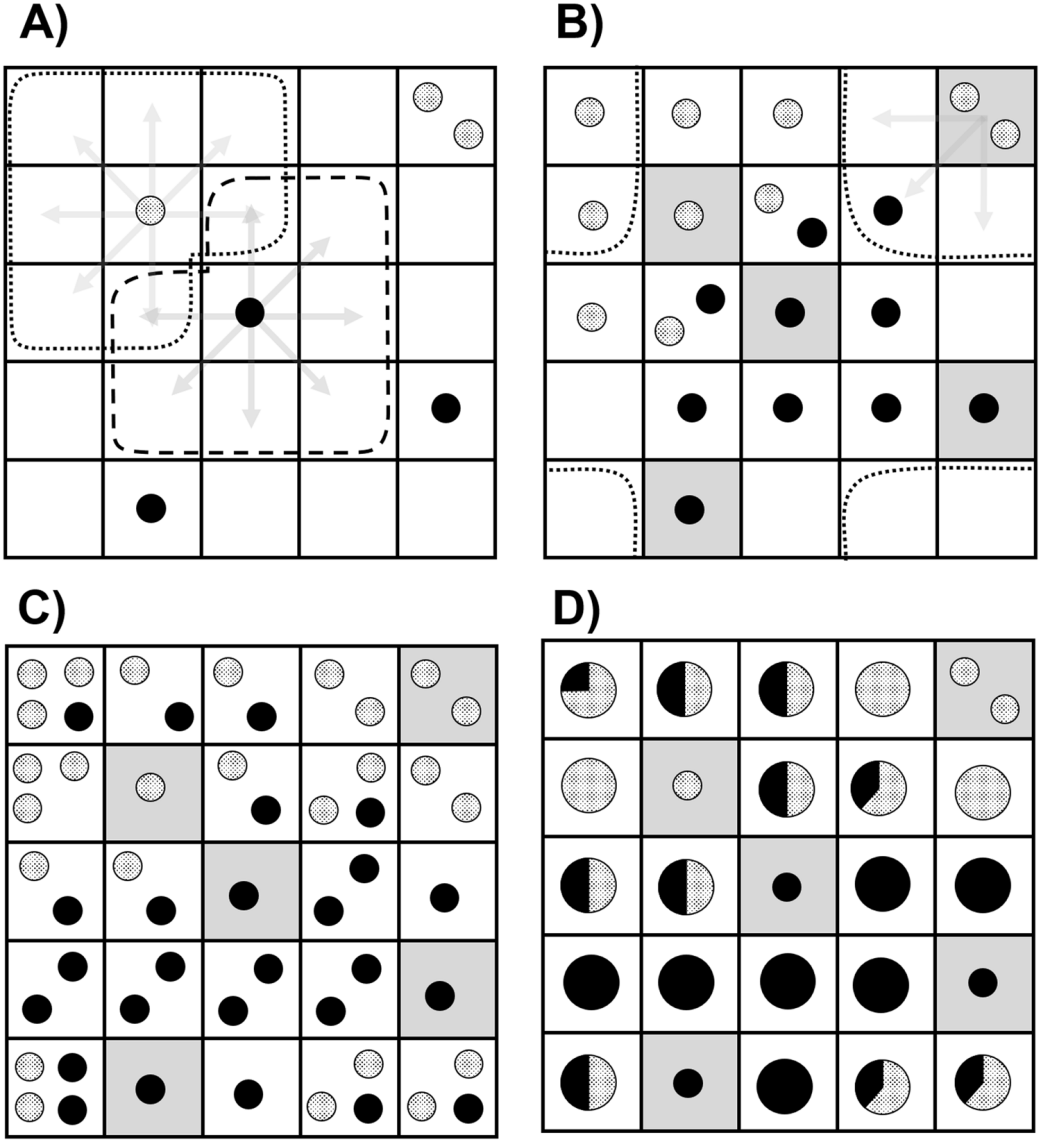
Graphical representation of the probability expansion algorithm proposed for SOM mapping. Considering a binary classification problem (classes are represented as solid and pointed dots) the expansion algorithm proposed undertake the following steps: (**A**) After SOM mapping into a 5 × 5 neuron space, each filled position transfers its content to the adjacent coordinates (grey arrows), excluding the filled positions of the training set. (**B**) This procedure also affects boundary neurons since these positions are interrelated, generating a toroidal shaped space. Note that grey cells account for the original occupied neurons and they are not affected by the expansion algorithm. (**C**) The contributions are added up at every position. (**D**) Final probabilities of original unoccupied neurons are therefore calculated according to the number of examples within each coordinate.

After SOM projection, labelled data partially occupy a toroidal-shaped space (usually represented as a plane). The probability expansion algorithm is therefore applied to fill all the SOM cells by transferring the label of projected examples to all their unoccupied adjacent positions. After performing this operation, the probability of each position to belong to one class is obtained as the percentage after adding all the contributions (Fig. 1).

**Figure 2:**
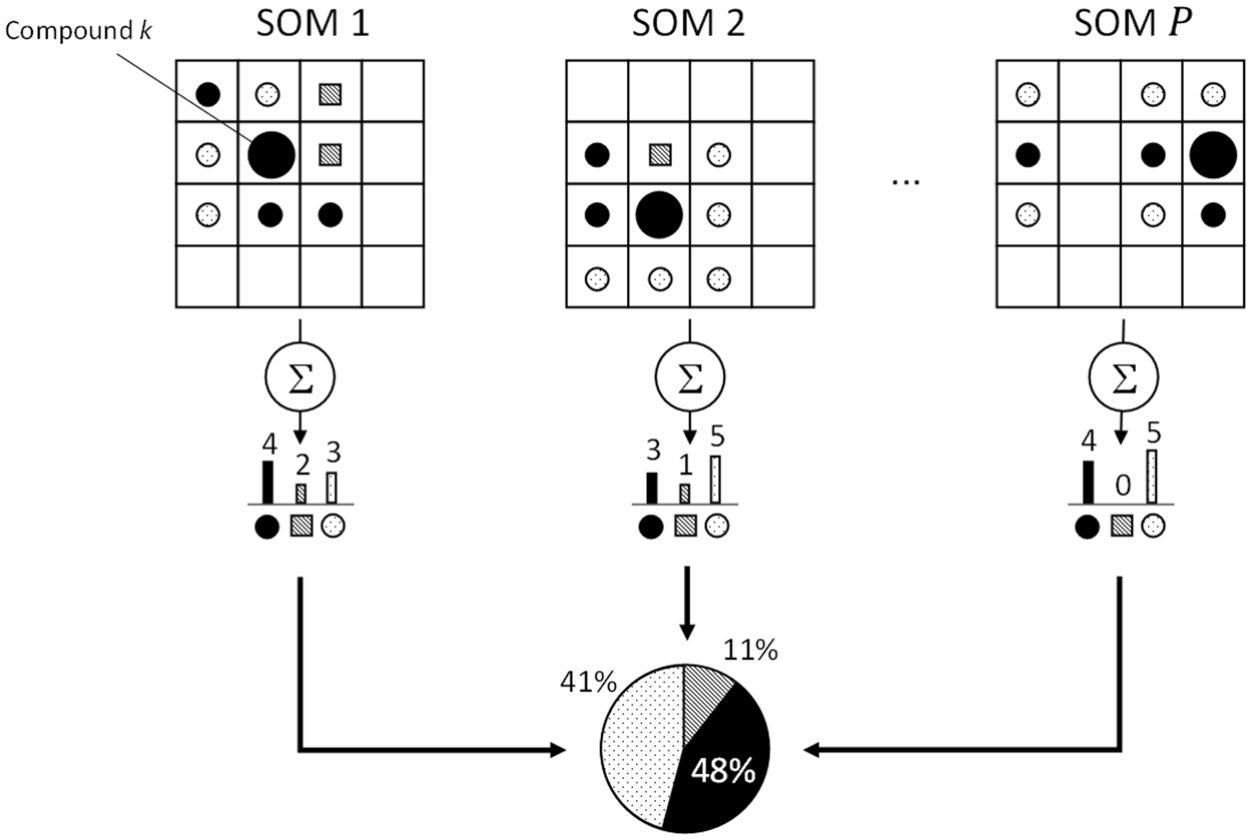
Schematic representation of the algorithm implemented to improve predictions in the boundaries. The neighbours of a given compound (k) can be different at each SOM run. With this algorithm, we would like to identify those coordinates that tend to locate in boundaries. The class of every neighbour is added and the total distribution is averaged over all calculated SOMs. Resulting probabilities were therefore used as prior probabilities in the expansion algorithm.

Due to the random nature of unsupervised learning, consecutive runs may lead to different results, hampering the generalization of results. Thereby, the interpretation of the area surrounding a molecule which tends to be enclosed within a class may be different from those molecules that mostly remain in boundaries. However, similarity in the feature space should be conserved in a SOM^25^ (i.e. similar inputs are located close to each other). In order to determine which partner a molecule prefers, we statistically determined if a given training example tends to behave as one member of its class, or if it is usually located within another label. This value is obtained by comparing the SOM projection of a single training set starting P-times from different random points. and considering the neighbours of each example throughout all repeats (Fig. 2). The information gathered was used as prior probability in the expansion algorithm.

Results allowed to divide the projected space into the most probable regions to obtain a given class. The usefulness of this method relies on being able to distinguish the true positives from the dubious predictions (that contain a non-zero probability to belong to two or more classes). Thus, an example is considered as a good prediction if one of its probabilities is greater than a given threshold, otherwise it is classified as a non-conclusive prediction. Consequently, we can control the confidence level of the predictions by varying the threshold value and enriching the active substrate selection at the expense of removing dubious points. In the context of ABC-transporter, the method presented herein would be able to predict the probability of a compound for being transported.

Since the original SOM algorithm highly depends on the number of training examples, minority classes in imbalanced data sets might be absorbed by the majority class. For this reason, we have evaluated the effect of under- and over-sampling on SOM mapping when conducting the classification of ABC-transporters substrates. A 10-fold cross-validation has been applied in all calculations to evaluate the data set independency.

### Software

Classification methods, including the corresponding cross-validation were performed in the Rapidminer 5.0 software (RM)^26^. Under- and over-sampling algorithms were integrated in the workflow by means of RM R extension. The R software was used to over-sampling using the ‘smote’ function available at ‘DMwR’ package^27^ and for random under-sampling, using the ‘sample’ function (‘mlr’ package)^28^.

ChemGPS descriptors were calculated for all compounds included in the database using the ChemGPS webserver^18^, whereas Shannon Entropy Descriptors (SHED) were calculated using MOE (downloadable from SVL Exchange repository). Auto-Associative Neural Networks and Consensus SOM functions were implemented using R scripting.

### Data availability

The datasets analysed during the current study are available in public repositories (see Data Sets section).

## Results and Discussion

### Validation of the CSOM algorithm

Four benchmark data sets were used to validate the effectiveness of the proposed CSOM methodology in comparison with a k-NN classifier.10-fold cross-validation was applied to evaluate the prediction ability in all models.

**Figure 3:**
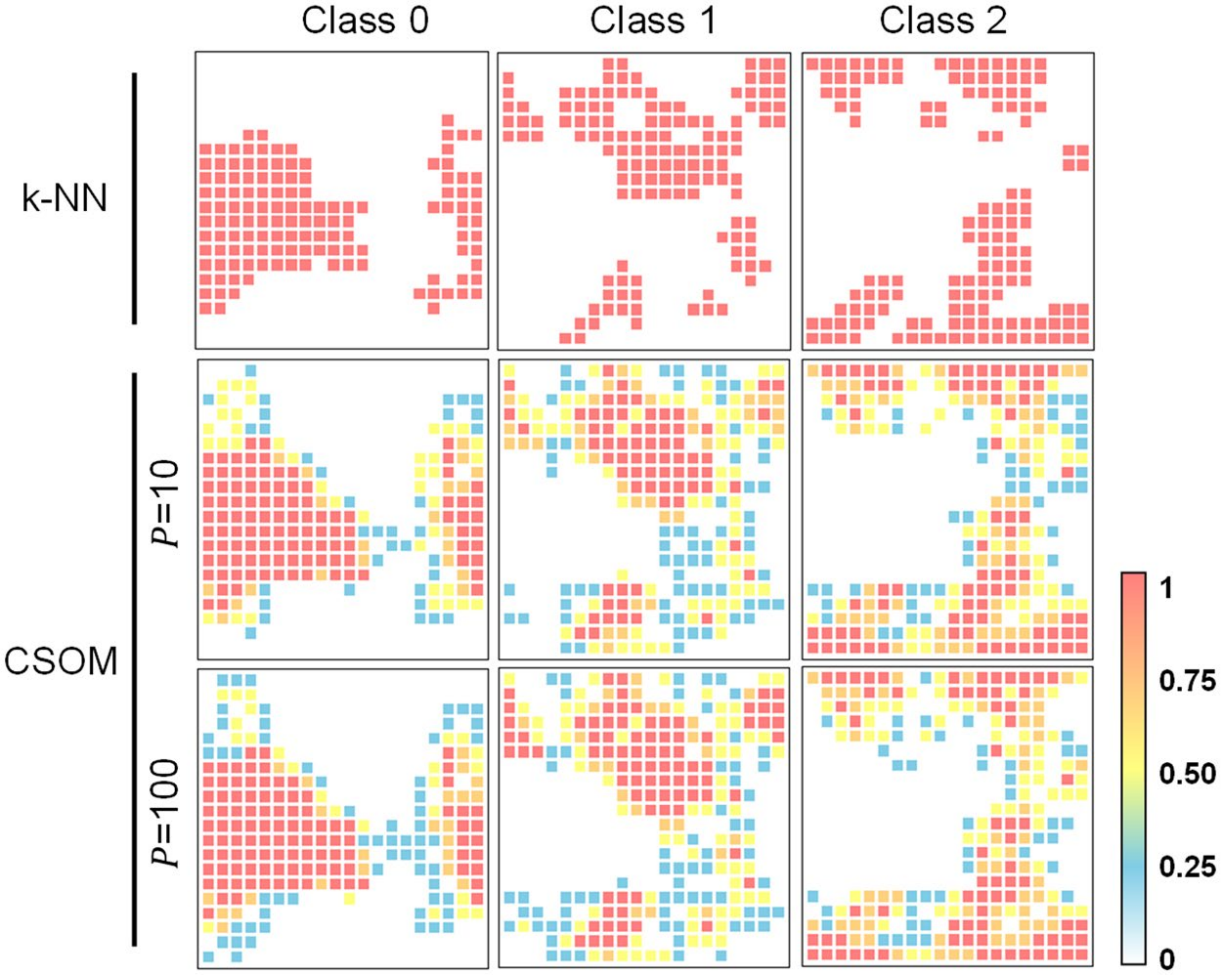
Differences in class probabilities when mapping wines data set in a 20 × 20 SOM using k-NN and CSOM algorithms. Higher P value refer to areas with higher class probability.

The CSOM expansion algorithm described above is based on the combination of the gathered information from a P-times repeated SOM. Preliminary results suggest that P~10 is enough to ensure the convergence of class probabilities (Fig. 3).

All calculations were conducted by defining 50 training rounds, a decaying learning rate from 2.0 to 0.01 and an initial adaptation radius of 20.0. In the case of the Yeast data set, the SMOTE algorithm was used to increase the number of examples in the minority classes.

**Figure 4:**
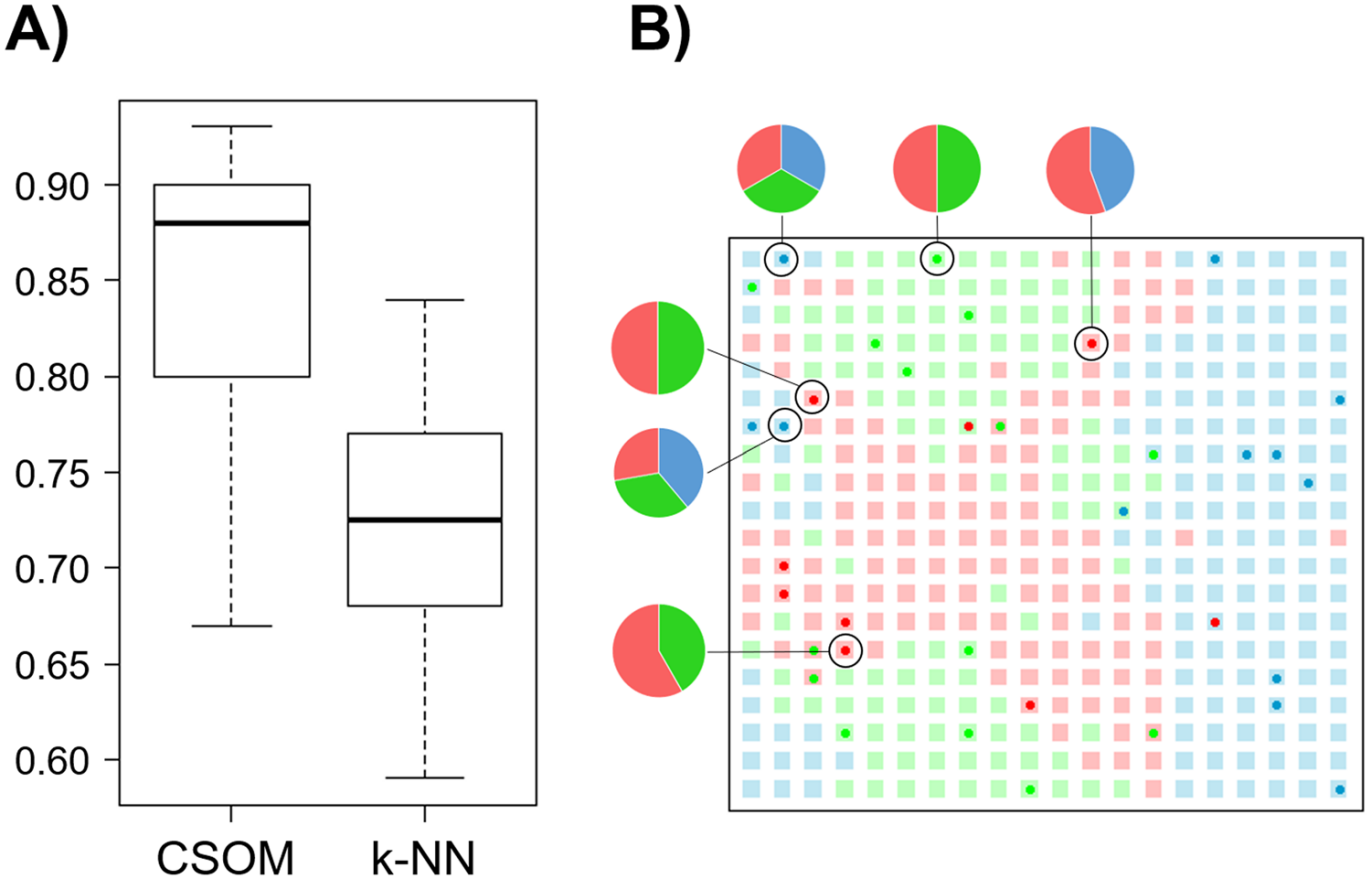
(**A**) Distribution of the accuracies obtained in 10-fold cross-validation by applying k-NN and CSOM algorithms on wines database. (**B**) Example of a mapped CSOM in which the probability of the six non-conclusive examples reported by the algorithm are shown. Interestingly, true class is the one with the higher probability in all the examples, although it is not enough to achieve the threshold value (t > 0.5).

Results show that the mean recall obtained by CSOM is statistically greater than the one obtained with the k-NN classifier. (Fig. 4).

In all cases the CSOM strategy leads to better classification results than k-NN. Paired Student’s t-test was performed for the statistical analysis of averaged mean recall values obtained by both methods. Results evince the existence of a statistical significant difference between averages (p value < 0.05) for all data sets with the exception of the New Thyroid data set, in which both methods led to good results (Table 3). Nevertheless, the identification of non-conclusive points in the SOM space becomes the main advantage of the CSOM algorithm, allowing to reduce misclassifications and to increase the mean recall.

**Table 3:** Results obtained using k-NN and CSOM applying 10-fold cross-validation.

Database	Method	Acc.	Mean Recall	F	p-value
Wines	CSOM (t = 0.6)	0.86	0.84	0.86	0.008
	k-NN	0.77	0.72	0.73	
Yeast	CSOM (t = 0.5)	0.58	0.52	0.52	0.005
	k-NN	0.50	0.45	0.45	
New Thyroid	CSOM (t = 0.5)	0.93	0.90	0.91	0.17
	k-NN	0.90	0.85	0.86	
Cars	CSOM (t = 0.7)	0.91	0.82	0.83	0.006
	k-NN	0.88	0.74	0.75	

The effect of the threshold values (t) were studied individually for each data set (see supporting information, Fig. S2).

Results show that the mean recall obtained by CSOM is statistically greater than the one obtained with k-NN. Thus, we hypothesized that CSOM could be considered for the classification of ABC transporter substrates.

### Classification of ABC-transporter substrates

**Figure 5:**
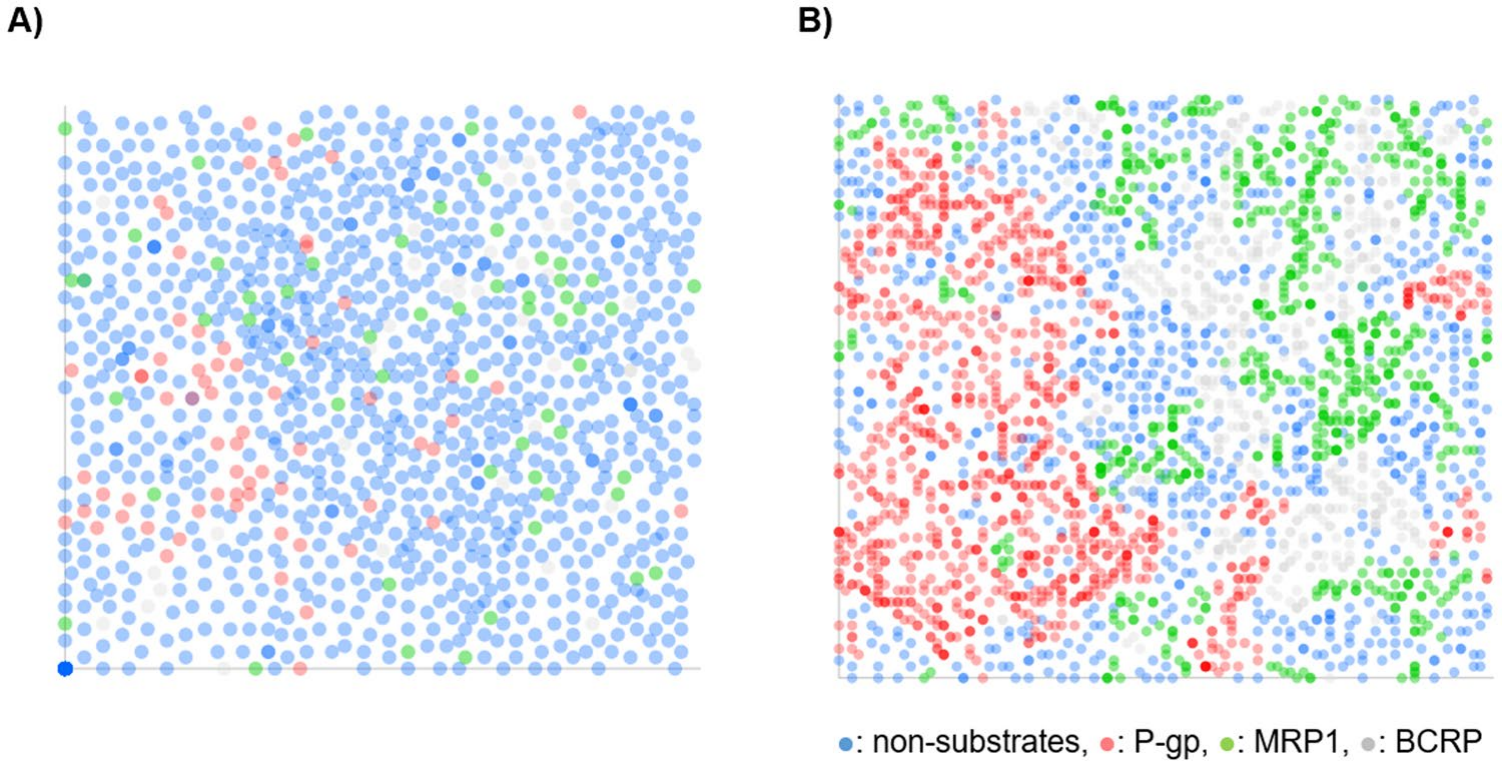
SOM projection of the original data set using ChemGPS descriptors (**A**) in contrast to the SMOTE data set (**B**) on 100 × 100 SOM with a variable adaptation radius from 10 to 1 in 100 iterations.

The data set including ABC-transporter substrates was projected into the SOM space by applying the molecular descriptors previously commented. Due to data imbalance, the SMOTE algorithm became mandatory to keep the data clustered (Fig. 5).

The CSOM algorithm was applied for the classification of multi-labelled ABC-transporter substrates by performing a 10-fold cross-validation for each set of molecular descriptors considered. Considering the heterogeneity of the dataset, we set the threshold value to 0.9 in order to increase the number of reliable classifications, at expense of increasing the number of non-conclusive results.

The results obtained by applying a k-NN classifier (Table 4) followed the same trend as benchmark analysis previously described. CSOM was able to improve k-NN results by identifying those substrates with higher probability to be misclassified. Among all sets of descriptors tested, AANN and DD17 reached the highest mean recall values with comparable F measure values, leading to a good compromise between mean recall and mean prediction.

**Table 4:** 10-fold cross validation results obtained in the classification of ABC-transporter substrates.

Descriptors	Classification Method	Accuracy	Mean Recall	F-measure	% out
ChemGPS	**SOM+CSOM**	**0.75**	**0.48**	**0.45**	**4.2**
	SOM+k-NN	0.69	0.45	0.40	
	k-NN	0.68	0.45	0.41	
	RF	0.73	0.43	0.41	
SHED	**SOM+CSOM**	**0.72**	**0.44**	**0.41**	**4.9**
	SOM+k-NN	0.66	0.42	0.38	
	k-NN	0.66	0.44	0.39	
	RF	0.74	0.42	0.41	
DD17	**SOM+CSOM**	**0.77**	**0.51**	**0.48**	**6.3**
	SOM+k-NN	0.69	0.44	0.40	
	k-NN	0.65	0.45	0.40	
	RF	0.77	0.45	0.44	
AANN	**SOM+CSOM**	**0.73**	**0.51**	**0.46**	**6.5**
	SOM+k-NN	0.65	0.41	0.37	
	k-NN	0.65	0.45	0.40	
	RF	0.71	0.43	0.41	

The use of SOM projection combined with CSOM and k-NN are compared with those obtained directly with the original dataset. For the sake of clarity, only results regarding k-NN and Random Forest (RF) are presented. In all cases SMOTE oversampling was applied. SOM topology was fixed to 100 × 100, initial adaptation radius and CSOM threshold were set to 20 and 0.9, correspondingly. The percentage of non-conclusive examples (% out) are shown when the CSOM approach is used.

To ascertain the goodness of the results obtained, we considered a total of 7 alternative classification methods (including supervised learners such as back-propagation neural networks or support vector machines). We applied these methods on the ABC-transporter data set, defining the same sets of descriptors and evaluating the use of over-sampling, under-sampling and cost-sensitive approaches to handle data imbalance (results are shown in the supporting information, Fig. S3). CSOM results were not only comparable with the best result achieved with alternative methods (i.e. Random Forest), they also showed the best F-measure value.

In contrast to CSOM, the best result obtained by k-NN corresponds to the ChemGPS descriptors (0.77 accuracy, 0.39 mean recall, 0.39 F-measure). The improvement obtained by CSOM was due to the identification of non-conclusive examples (i.e. 6.3% of the test set).

**Figure 6:**
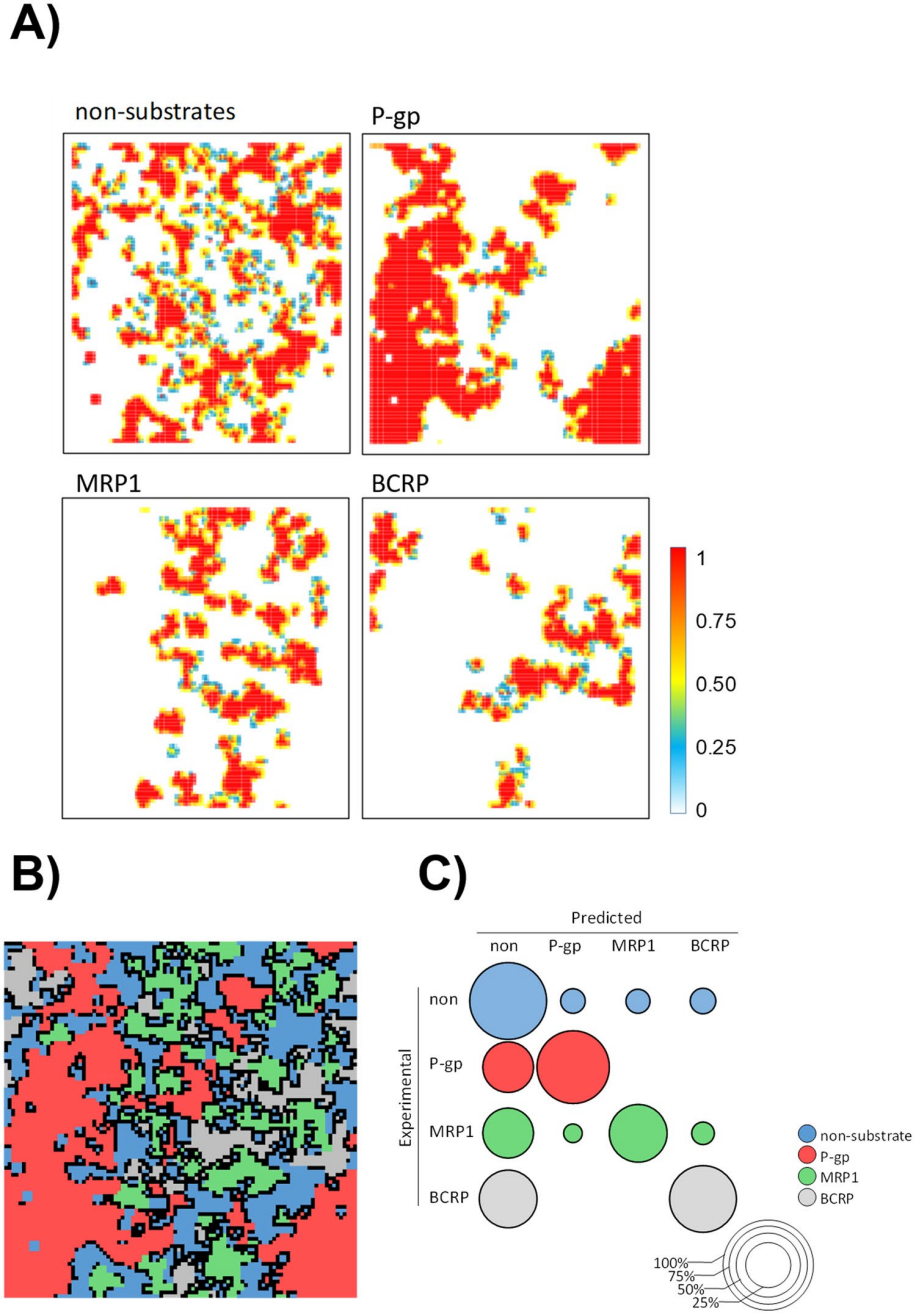
Distribution of the prior probabilities (calculated by the CSOM algorithm) on SOM coordinates for every kind of ABC-transporter substrates in the training set (**A**). The combination of these probabilities allowed to obtain the mapped SOM in which black dots account for non-conclusive coordinates (**B**). Sum of calculated probabilities for the test set examples, organized as a confusion matrix. The size of rounded shape accounts for the probability of obtaining the corresponding predicted class (**C**).

Finally, we studied the information gathered in the minority probabilities and evaluated their influence on the classification results. For this reason, the CSOM methodology was applied at a test set randomly selected from the original data set by stratified sampling, which included 102 non-substrates, and 7 P-gp, 7 MRP1 and 4 BCRP substrates. The rest of the data set was used as the training set (i.e. 1084 examples). Data was projected into the SOM space using the CSOM clustering algorithm (50 iterations). Probability density generated for non-substrate coordinates are largely scattered within the space due to the high disparity in molecular structures included in this class (Fig. 6A).

Non-conclusive examples were already in the training set. As expected, these positions were located outlining boundary regions (Fig. 6B). After mapping the test set, we obtained an accuracy of 0.80, a mean recall of 0.64, and a F measure of 0.58. 29 examples were classified as non-conclusive. Adding all probabilities obtained for the test set (Fig. 6C) showed a clear trend to guess the correct class. Although the effect of data imbalance fosters the presence of a moderate probability to classify any of the ABC-transporter substrates as a non-substrate, there were no miss-classifications entailing two ABC-transporters.

## Conclusions

Self-Organizing Maps have been extensively applied for classification purposes. In this study we evaluated the use of this technique for substrate classification for three types of ABC transporters (i.e. P-glycoprotein, MRP1, and BCRP). Unfortunately, the structure disparity of non-substrates led to a highly imbalanced data set. This situation hindered getting acceptable mean recall values when SOMs were combined with traditional clustering algorithms such as k-NN. Therefore, we proposed the CSOM expansion algorithm, which is able to label every SOM coordinate to a particular class, according to a statistical probability estimated by weighted voting. Prior probabilities are calculated by combining the information gathered from the sequentially repetition of a training SOM, randomly initialized at every iteration. The best results were obtained by applying CSOM methodology on the DD17 set (containing 17 molecular descriptors adapted from ABC-transporter substrates binary classifiers reported in literature). The obtained accuracy was in agreement with previously reported results for single ABC-transporter classification^16^, although averaged mean recall and F measures were more modest due to data imbalance (0.77 accuracy, 0.51 mean recall, 0.48 F measure in 10-fold cross-validation). However, values are comparable to those obtained with supervised learning. To the best of our knowledge, this is the first efficient unsupervised approach, which is - in combination with oversampling techniques - able to handle multi-labelled data and provides similar results than random forest. Moreover, the major asset of CSOM relies on its ability of enriching compound selections, discarding those examples with higher probability to be misclassified and reducing uncertainty.

Having a multi-labelled classification method available for P-glycoprotein, MRP1 and BCRP substrates can very useful for evaluating the pharmacological profile of drug candidates against these ABC-transporters. This information can be further used to predict ADMET properties and anticancer drug resistance.

## Electronic supplementary material

Supporting information
