# Supplementary material for: Predicting drug resistance related to ABC transporters using unsupervised Consensus Self-Organizing Maps
Source: Sci Rep. 2018 May 1;8:6803. doi: 10.1038/s41598-018-25235-9 (PMC5931609; doi:10.1038/s41598-018-25235-9)
Supplement: Supplementary file 1 — Supporting information [file 41598_2018_25235_MOESM1_ESM.pdf]

Predicting drug resistance related to ABC transporters using unsupervised Consensus Self-Organizing Maps

Roger Estrada-Tejedor[1,*] and Gerhard F. Ecker[2]

[1] IQS School of Engineering – Universitat Ramon Llull, Via Augusta 390, 08017 Barcelona, Spain

[2] University of Vienna, Department of Pharmaceutical Chemistry, Althanstrasse 14, 1090 Vienna, Austria

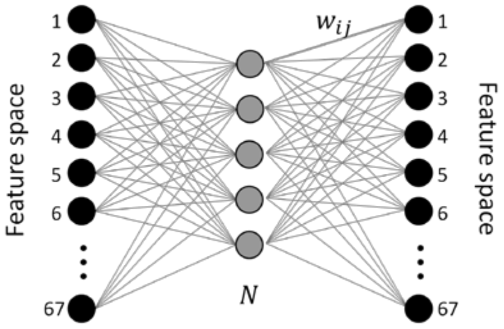

Figure S1. Graphical representation of the Auto-Associative Neural Network applied for reducing the feature space to N=10 variables.

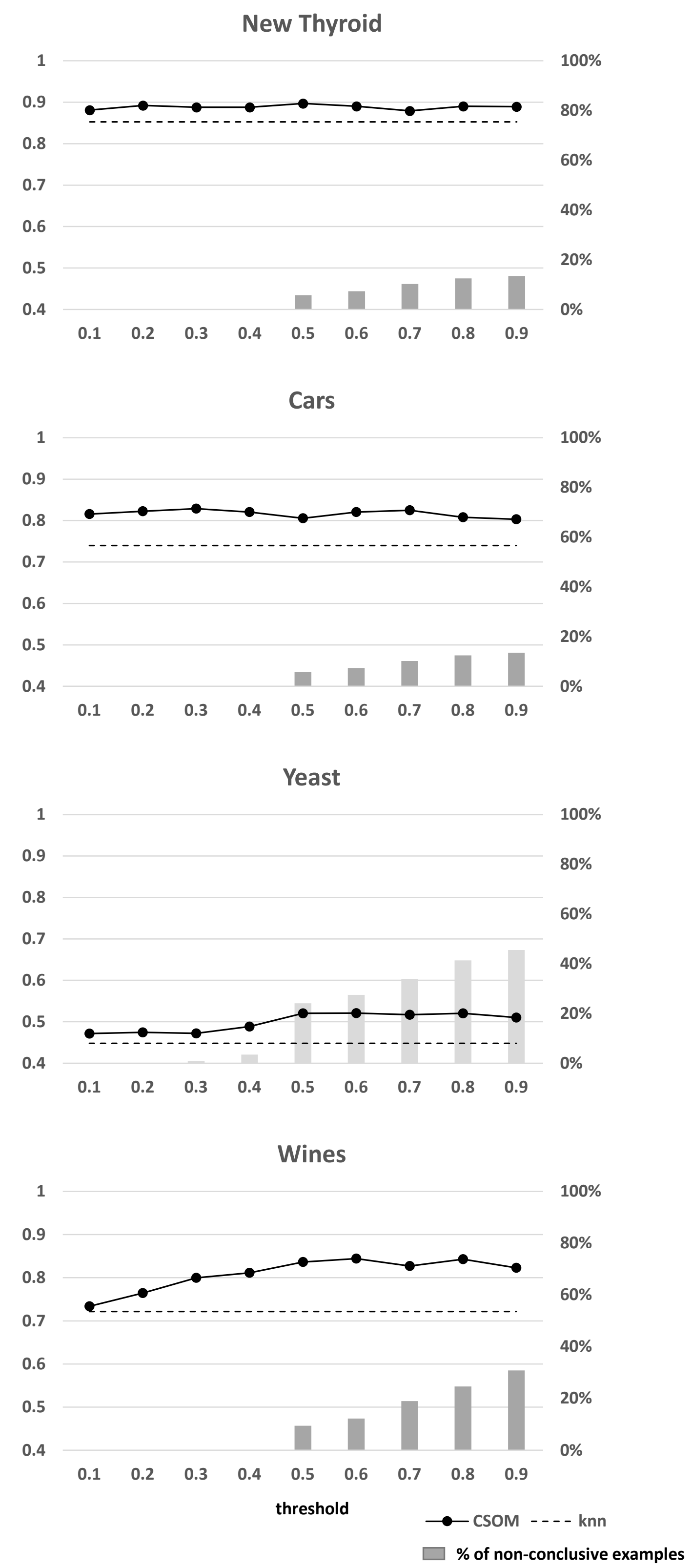

Figure S2. Effect of the threshold value defined in CSOM algorithm on the classification ability, quantified as the averaged Mean Recall (left-axis) in benchmark datasets. The number of examples considered as non-conclusive are also included as grey bars (right-axis). In all cases, the best threshold value is obtained between 0.5 and 0.7.

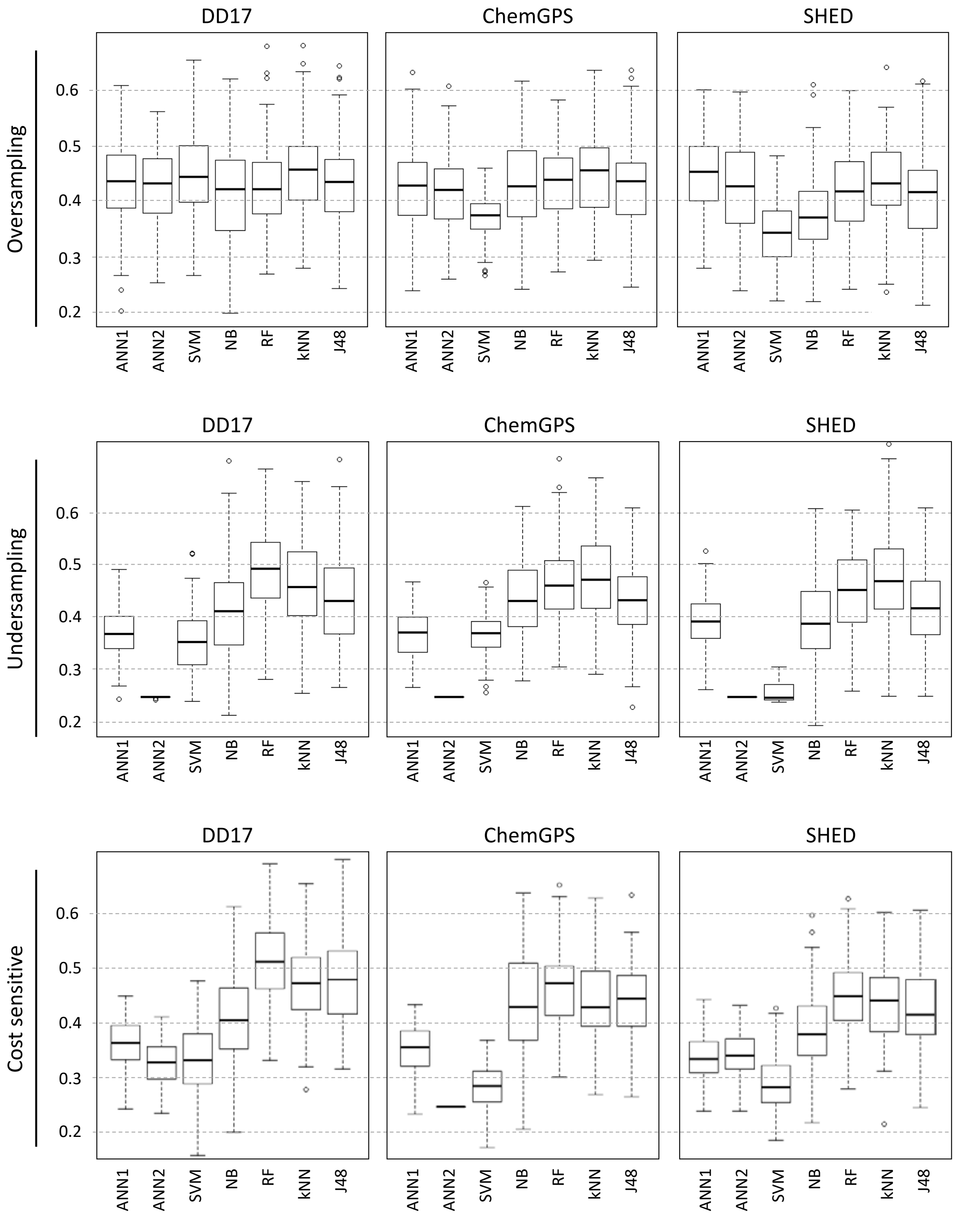

Figure S3. Preliminary results obtained by applying different classification methods including artificial neural networks (with 1 or 2 hidden layers, ANN1 and ANN2 correspondingly), Support Vector Machines (SVM), Naïve Bayes (NB), Random Forest (RF), k-Nearest Neighbour (k-NN) and (J48). Only DD17, ChemGPS and SHED descriptors were initially considered. Box plots represent the averaged mean recall obtained (n=100). Test sets were randomly selected by stratified sampling (which account for the 10% of the dataset). The best model was obtained by applying a cost sensitive Random Forest, which present 0.66 accuracy, 0.39 mean prediction, 0.51 mean recall and 0.44 F-measure.
